# Bis(triethano­lamine)nickel(II) sulfate

**DOI:** 10.1107/S1600536809023046

**Published:** 2009-06-20

**Authors:** Hong-Xu Guo, Zi-Xian Du, Xi-Zhong Li

**Affiliations:** a Department of Chemistry and Environmental Science, Zhangzhou Normal University, Zhangzhou, Fujian 363000, People’s Republic of China; b Fujian Longyan Center for Disease Control & Prevention, Longyan, Fujian, 364000, People’s Republic of China

## Abstract

The title compound, [Ni(C_6_H_15_NO_3_)_2_]SO_4_, contains two triethano­lamine (TEA) ligands bound to an Ni^2+^ metal centre, which lies on a crystallographic inversion centre, and one sulfate anion located on a twofold rotation axis such that the asymmetric unit contains one-half molecule of the  cation and of the anion. The triethano­lamine ligands coordinate *via* each axial N atom and two of the three O atoms, while the third arm of the ligand has the hydroxyl group pointing away from the metal centre. The sulfate anions are hydrogen bonded to the coordinated hydroxyl groups and also to the free arm, forming a two-dimensional supra­molecular hydrogen-bonded network expanding parallel to (010).

## Related literature

For background to metal-ion-containing supra­molecular compounds, see: Venkataraman *et al.* (1995 ▶); Kepert & Rosseinsky (1999 ▶); Fujita *et al.* (1994 ▶). For magnetic materials, see: Kahn (1993 ▶). For other TEA compounds, see: Krabbes *et al.* (2000 ▶); Topcu *et al.* (2001 ▶); Ucar *et al.* (2004 ▶); Haukka *et al.* (2005 ▶). For similar structures, see: İçbudak *et al.* (1995 ▶); Yeşilel *et al.* (2004 ▶).
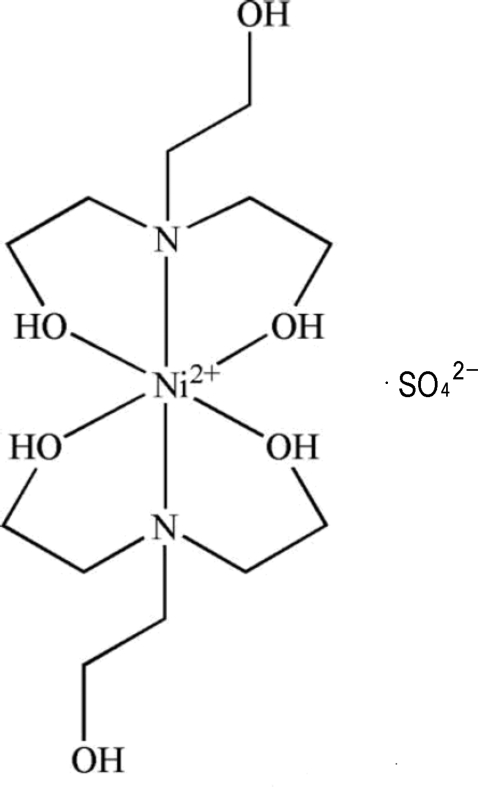


## Experimental

### 

#### Crystal data

[Ni(C_6_H_15_NO_3_)_2_]SO_4_
*M* *_r_* = 453.15
Monoclinic, 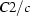

*a* = 10.316 (2) Å
*b* = 11.234 (2) Å
*c* = 15.498 (3) Å
β = 90.04 (3)°
*V* = 1796.0 (6) Å^3^
*Z* = 4
Mo *K*α radiation
μ = 1.25 mm^−1^
*T* = 293 K
0.41 × 0.21 × 0.11 mm

#### Data collection

Siemens SMART CCD area-detector diffractometer
Absorption correction: multi-scan (*SADABS*; Sheldrick, 1996 ▶) *T*_min_ = 0.741, *T*_max_ = 0.879
8633 measured reflections
2042 independent reflections
1905 reflections with *I* > 2σ(*I*)
*R*_int_ = 0.029

#### Refinement

*R*[*F* ^2^ > 2σ(*F* ^2^)] = 0.030
*wR*(*F* ^2^) = 0.085
*S* = 1.02
2042 reflections
120 parameters
3 restraints
H-atom parameters constrained
Δρ_max_ = 0.66 e Å^−3^
Δρ_min_ = −0.65 e Å^−3^

### 

Data collection: *SMART* (Siemens, 1994 ▶); cell refinement: *SAINT* (Siemens, 1994 ▶); data reduction: *SAINT*; program(s) used to solve structure: *SHELXTL* (Sheldrick, 2008 ▶); program(s) used to refine structure: *SHELXTL*; molecular graphics: *SHELXTL*; software used to prepare material for publication: *SHELXTL*.

## Supplementary Material

Crystal structure: contains datablocks I, global. DOI: 10.1107/S1600536809023046/pk2167sup1.cif

Structure factors: contains datablocks I. DOI: 10.1107/S1600536809023046/pk2167Isup2.hkl

Additional supplementary materials:  crystallographic information; 3D view; checkCIF report

## Figures and Tables

**Table 1:** Hydrogen-bond geometry (Å, °)

*D*—H⋯*A*	*D*—H	H⋯*A*	*D*⋯*A*	*D*—H⋯*A*
O1—H1*C*⋯O5^i^	0.82	2.19	2.663 (2)	117
O2—H2*C*⋯O4^ii^	0.82	2.28	2.6227 (19)	106
O3—H3*C*⋯O5	0.82	2.00	2.818 (2)	174
C3—H3*B*⋯O3	0.97	2.26	3.055 (3)	139

Symmetry codes: (i) 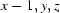; (ii) 
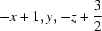
.

**Fig. 1.**
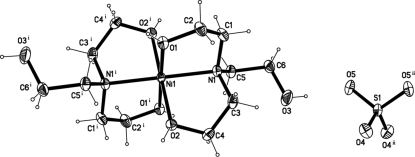
View of the structure showing the atom-labelling scheme. Displacement ellipsoids are drawn at the 35% probability level; H-atoms are shown as small spheres of arbitrary radius. (Symmetry codes: i: -x+1/2, -y+3/2,-z+2; ii: -x+2, y, -z+3/2)

**Fig. 2.**
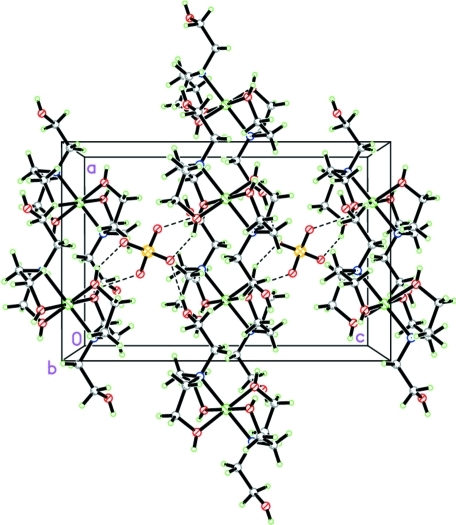
View of the 2-D hydrogen-bonded network in the packing of the title compound. The packing is viewed along the b axis; O-H···O and C—H···O interactions are shown as dashed lines.

## supplementary crystallographic information

### Comment

Many workers from a variety of scientific disciplines are interested in the crystal design and engineering of multidimensional arrays and networks containing metal ions as nodes. Metal-ion containing supramolecular structures can be used as zeolite-like materials (Venkataraman *et al.*, 1995; Kepert & Rosseinsky, 1999), catalysts (Fujita *et al.*, 1994) or magnetic materials (Kahn, 1993). Triethanolamine (TEA) is a good potential ligand to the incorporation of metals into metal-ion containing supramolecular framework, and many compounds constructed from TEA have been reported in the last decade (Krabbes *et al.*, 2000; Topcu *et al.*, 2001; Ucar *et al.*, 2004; Haukka *et al.*, 2005). In this work we employed TEA and NiSO_4_ to produce a novel complex, [Ni(C_6_H_15_NO_3_)_2_]SO_4_(I).

A view of the title compound and its numbering scheme are shown in Fig. 1. The crystal structure consists of a complex cation and one sulfate anion. In the cation, the Ni^II^ ion lies on a centre of symmetry, sandwiched by two bulky TEA ligands. Each TEA acts as a tridentate ligand through two of the three hydroxyl O atoms and the amine N atom, resulting in a six-coordinate Ni^II^ ion similar to those observed for the Ni complex of TEA with chloride (İçbudak *et al.*, 1995), saccharine (Topcu *et al.*, 2001), acetate (Krabbes *et al.*, 2000) and squarate (Yeşilel *et al.*, 2004).

The coordination geometry around the Ni^II^ ion is that of a distorted octahedron. The hydroxyl O atoms of two TEA ligands form the equatorial plane of the octahedral geometry, while atoms N1 and N1^i^ are placed in axial positions (symmetry code as in Fig. 1). In the complex, Ni—O distances are in the range 2.0762 (13)–2.0768 (12)Å and the Ni—N distance is 2.1072 (16) Å, while the bond angles at Ni range from 82.30 (5)° to 97.70 (6)°.

In the crystal structure, classical intermolecular O—H···O hydrogen bonds are observed (Table 1), which link the cations and one sulfate anion into a two-dimensional hydrogen-bonded network and stabilize the crystal packing (Fig. 2). Each cation is bonded to four SO_4_^2-^ anions, which in turn link four cations through O—H···O hydrogen bonding interactions to form a 2-D hydrogen bonding network. In addition, there is extensive hydrogen bonding between the –CH_2_ and the uncoordinated hydroxyl O atoms, with C···O interatomic distances of 3.055 (3) Å.

### Experimental

NiSO_4_.7H_2_O (0.5257 g, 2 mmol) was dissolved in 10 ml water and the pH was adjusted to 7 with triethanolamine. Blue crystals of separated from the filtered solution at room temperature over several days.

### Refinement

All H atoms bound to carbon were refined using a riding model with C—H = 0.97Å and *U*_iso_(H) = 1.2*U*_eq_(C). Three hydroxy H atoms were located in a difference map and included with O—H distance constraints of 0.82 Å and with *U*_iso_(H) = 1.5*U*_eq_(O).

### Crystal data

**Table:**

[Ni(C_6_H_15_NO_3_)_2_]SO_4_	*F*(000) = 960
*M**_r_* = 453.15	*D*_x_ = 1.676 Mg m^−^^3^
Monoclinic, *C*2/*c*	Mo *K*α radiation, λ = 0.71073 Å
Hall symbol: -C 2yc	Cell parameters from 8633 reflections
*a* = 10.316 (2) Å	θ = 3.6–27.5°
*b* = 11.234 (2) Å	µ = 1.25 mm^−^^1^
*c* = 15.498 (3) Å	*T* = 293 K
β = 90.04 (3)°	Block, blue
*V* = 1796.0 (6) Å^3^	0.41 × 0.21 × 0.11 mm
*Z* = 4	

### Data collection

**Table:**

Siemens SMART CCD area-detector diffractometer	2042 independent reflections
Radiation source: fine-focus sealed tube	1905 reflections with *I* > 2σ(*I*)
graphite	*R*_int_ = 0.029
ω scans	θ_max_ = 27.5°, θ_min_ = 3.6°
Absorption correction: multi-scan (*SADABS*; Sheldrick, 1996)	*h* = −13→13
*T*_min_ = 0.741, *T*_max_ = 0.879	*k* = −14→14
8633 measured reflections	*l* = −20→18

### Refinement

**Table:**

Refinement on *F*^2^	Primary atom site location: structure-invariant direct methods
Least-squares matrix: full	Secondary atom site location: difference Fourier map
*R*[*F*^2^ > 2σ(*F*^2^)] = 0.030	Hydrogen site location: inferred from neighbouring sites
*wR*(*F*^2^) = 0.085	H-atom parameters constrained
*S* = 1.02	*w* = 1/[σ^2^(*F*_o_^2^) + (0.053*P*)^2^ + 2.3273*P*] where *P* = (*F*_o_^2^ + 2*F*_c_^2^)/3
2042 reflections	(Δ/σ)_max_ < 0.001
120 parameters	Δρ_max_ = 0.66 e Å^−^^3^
3 restraints	Δρ_min_ = −0.65 e Å^−^^3^

### Special details

**Table:**

Geometry. All e.s.d.'s (except the e.s.d. in the dihedral angle between two l.s. planes) are estimated using the full covariance matrix. The cell e.s.d.'s are taken into account individually in the estimation of e.s.d.'s in distances, angles and torsion angles; correlations between e.s.d.'s in cell parameters are only used when they are defined by crystal symmetry. An approximate (isotropic) treatment of cell e.s.d.'s is used for estimating e.s.d.'s involving l.s. planes.
Refinement. Refinement of *F*^2^ against ALL reflections. The weighted *R*-factor *wR* and goodness of fit *S* are based on *F*^2^, conventional *R*-factors *R* are based on *F*, with *F* set to zero for negative *F*^2^. The threshold expression of *F*^2^ > σ(*F*^2^) is used only for calculating *R*-factors(gt) *etc*. and is not relevant to the choice of reflections for refinement. *R*-factors based on *F*^2^ are statistically about twice as large as those based on *F*, and *R*- factors based on ALL data will be even larger.

### Fractional atomic coordinates and isotropic or equivalent isotropic displacement parameters (Å^2^)

**Table:**

	*x*	*y*	*z*	*U*_iso_*/*U*_eq_	
Ni1	0.2500	0.7500	1.0000	0.01765 (12)	
N1	0.40821 (14)	0.71956 (14)	0.91696 (9)	0.0218 (3)	
O1	0.15355 (12)	0.64708 (12)	0.90906 (8)	0.0255 (3)	
H1C	0.0790	0.6254	0.8997	0.038*	
O2	0.22752 (12)	0.90445 (11)	0.92788 (8)	0.0242 (3)	
H2C	0.1762	0.9553	0.9113	0.036*	
O3	0.70194 (16)	0.79446 (17)	0.87607 (13)	0.0512 (5)	
H3C	0.7766	0.7847	0.8594	0.077*	
O4	0.89097 (13)	0.89514 (14)	0.72175 (9)	0.0351 (3)	
O5	0.95708 (14)	0.74424 (13)	0.82252 (10)	0.0337 (3)	
C1	0.37671 (18)	0.60337 (18)	0.87593 (13)	0.0303 (4)	
H1A	0.3897	0.5400	0.9176	0.036*	
H1B	0.4354	0.5898	0.8281	0.036*	
C2	0.23847 (19)	0.59917 (19)	0.84339 (12)	0.0317 (4)	
H2A	0.2307	0.6456	0.7909	0.038*	
H2B	0.2143	0.5176	0.8305	0.038*	
C3	0.40912 (18)	0.81992 (18)	0.85289 (12)	0.0293 (4)	
H3A	0.3573	0.7986	0.8030	0.035*	
H3B	0.4971	0.8347	0.8337	0.035*	
C4	0.35485 (18)	0.93041 (17)	0.89416 (12)	0.0288 (4)	
H4A	0.4114	0.9565	0.9405	0.035*	
H4B	0.3493	0.9938	0.8518	0.035*	
C5	0.53313 (17)	0.71242 (18)	0.96482 (12)	0.0265 (4)	
H5A	0.5251	0.6503	1.0080	0.032*	
H5B	0.5454	0.7870	0.9953	0.032*	
C6	0.65530 (18)	0.68791 (18)	0.91236 (14)	0.0321 (4)	
H6A	0.7212	0.6534	0.9493	0.039*	
H6B	0.6360	0.6314	0.8668	0.039*	
S1	1.0000	0.82070 (6)	0.7500	0.02289 (15)	

### Atomic displacement parameters (Å^2^)

**Table:**

	*U*^11^	*U*^22^	*U*^33^	*U*^12^	*U*^13^	*U*^23^
Ni1	0.01652 (17)	0.02186 (18)	0.01456 (17)	0.00161 (10)	0.00033 (11)	0.00006 (10)
N1	0.0191 (7)	0.0284 (7)	0.0179 (7)	0.0031 (6)	0.0008 (5)	0.0003 (6)
O1	0.0212 (6)	0.0335 (7)	0.0218 (6)	−0.0002 (5)	−0.0017 (5)	−0.0050 (5)
O2	0.0245 (6)	0.0257 (6)	0.0225 (6)	0.0044 (5)	−0.0008 (5)	0.0038 (5)
O3	0.0272 (8)	0.0572 (10)	0.0693 (12)	0.0037 (7)	0.0176 (8)	0.0185 (9)
O4	0.0287 (7)	0.0462 (8)	0.0303 (7)	0.0102 (6)	−0.0100 (6)	−0.0027 (6)
O5	0.0230 (7)	0.0524 (9)	0.0258 (7)	−0.0012 (5)	0.0023 (6)	0.0106 (6)
C1	0.0275 (9)	0.0335 (10)	0.0299 (9)	0.0064 (7)	0.0008 (7)	−0.0095 (8)
C2	0.0300 (10)	0.0393 (10)	0.0259 (9)	0.0030 (8)	−0.0013 (7)	−0.0127 (8)
C3	0.0249 (9)	0.0428 (11)	0.0201 (8)	0.0031 (7)	0.0028 (7)	0.0069 (7)
C4	0.0265 (9)	0.0304 (9)	0.0294 (9)	−0.0040 (7)	−0.0016 (7)	0.0081 (7)
C5	0.0208 (8)	0.0364 (9)	0.0223 (8)	0.0031 (7)	−0.0005 (7)	0.0024 (7)
C6	0.0221 (9)	0.0359 (10)	0.0383 (10)	0.0063 (7)	0.0015 (7)	−0.0019 (8)
S1	0.0172 (3)	0.0350 (3)	0.0165 (3)	0.000	−0.0021 (2)	0.000

### Geometric parameters (Å, °)

**Table:**

Ni1—O1^i^	2.0762 (13)	C1—C2	1.513 (3)
Ni1—O1	2.0762 (13)	C1—H1A	0.9700
Ni1—O2^i^	2.0768 (12)	C1—H1B	0.9700
Ni1—O2	2.0768 (12)	C2—H2A	0.9700
Ni1—N1	2.1072 (16)	C2—H2B	0.9700
Ni1—N1^i^	2.1072 (16)	C3—C4	1.505 (3)
N1—C1	1.488 (2)	C3—H3A	0.9700
N1—C5	1.489 (2)	C3—H3B	0.9700
N1—C3	1.502 (2)	C4—H4A	0.9700
O1—C2	1.447 (2)	C4—H4B	0.9700
O1—H1C	0.8200	C5—C6	1.525 (3)
O2—C4	1.444 (2)	C5—H5A	0.9700
O2—H2C	0.8200	C5—H5B	0.9700
O3—C6	1.407 (3)	C6—H6A	0.9700
O3—H3C	0.8200	C6—H6B	0.9700
O4—S1	1.4681 (14)	S1—O4^ii^	1.4681 (14)
O5—S1	1.4825 (14)	S1—O5^ii^	1.4825 (14)
			
O1^i^—Ni1—O1	180.0	O1—C2—H2A	109.9
O1^i^—Ni1—O2^i^	92.68 (5)	C1—C2—H2A	109.9
O1—Ni1—O2^i^	87.32 (5)	O1—C2—H2B	109.9
O1^i^—Ni1—O2	87.32 (5)	C1—C2—H2B	109.9
O1—Ni1—O2	92.68 (5)	H2A—C2—H2B	108.3
O2^i^—Ni1—O2	180.0	N1—C3—C4	109.61 (14)
O1^i^—Ni1—N1	97.70 (6)	N1—C3—H3A	109.7
O1—Ni1—N1	82.30 (5)	C4—C3—H3A	109.7
O2^i^—Ni1—N1	96.14 (5)	N1—C3—H3B	109.7
O2—Ni1—N1	83.86 (6)	C4—C3—H3B	109.7
O1^i^—Ni1—N1^i^	82.30 (5)	H3A—C3—H3B	108.2
O1—Ni1—N1^i^	97.70 (6)	O2—C4—C3	109.04 (15)
O2^i^—Ni1—N1^i^	83.86 (6)	O2—C4—H4A	109.9
O2—Ni1—N1^i^	96.14 (5)	C3—C4—H4A	109.9
N1—Ni1—N1^i^	180.0	O2—C4—H4B	109.9
C1—N1—C5	110.76 (15)	C3—C4—H4B	109.9
C1—N1—C3	112.18 (14)	H4A—C4—H4B	108.3
C5—N1—C3	111.34 (15)	N1—C5—C6	117.35 (15)
C1—N1—Ni1	103.56 (11)	N1—C5—H5A	108.0
C5—N1—Ni1	112.02 (11)	C6—C5—H5A	108.0
C3—N1—Ni1	106.69 (11)	N1—C5—H5B	108.0
C2—O1—Ni1	113.23 (10)	C6—C5—H5B	108.0
C2—O1—H1C	109.5	H5A—C5—H5B	107.2
Ni1—O1—H1C	137.3	O3—C6—C5	110.01 (16)
C4—O2—Ni1	105.21 (10)	O3—C6—H6A	109.7
C4—O2—H2C	109.5	C5—C6—H6A	109.7
Ni1—O2—H2C	145.3	O3—C6—H6B	109.7
C6—O3—H3C	109.5	C5—C6—H6B	109.7
N1—C1—C2	112.04 (15)	H6A—C6—H6B	108.2
N1—C1—H1A	109.2	O4^ii^—S1—O4	110.56 (13)
C2—C1—H1A	109.2	O4^ii^—S1—O5	109.46 (8)
N1—C1—H1B	109.2	O4—S1—O5	109.09 (9)
C2—C1—H1B	109.2	O4^ii^—S1—O5^ii^	109.09 (9)
H1A—C1—H1B	107.9	O4—S1—O5^ii^	109.46 (8)
O1—C2—C1	108.96 (15)	O5—S1—O5^ii^	109.18 (13)
			
O1^i^—Ni1—N1—C1	152.13 (11)	O1^i^—Ni1—O2—C4	72.48 (11)
O1—Ni1—N1—C1	−27.87 (11)	O1—Ni1—O2—C4	−107.52 (11)
O2^i^—Ni1—N1—C1	58.58 (11)	O2^i^—Ni1—O2—C4	−86 (100)
O2—Ni1—N1—C1	−121.42 (11)	N1—Ni1—O2—C4	−25.56 (11)
N1^i^—Ni1—N1—C1	−20 (100)	N1^i^—Ni1—O2—C4	154.44 (11)
O1^i^—Ni1—N1—C5	32.74 (13)	C5—N1—C1—C2	167.69 (16)
O1—Ni1—N1—C5	−147.26 (13)	C3—N1—C1—C2	−67.2 (2)
O2^i^—Ni1—N1—C5	−60.81 (13)	Ni1—N1—C1—C2	47.43 (17)
O2—Ni1—N1—C5	119.19 (13)	Ni1—O1—C2—C1	18.77 (19)
N1^i^—Ni1—N1—C5	−139 (100)	N1—C1—C2—O1	−45.3 (2)
O1^i^—Ni1—N1—C3	−89.34 (12)	C1—N1—C3—C4	143.10 (16)
O1—Ni1—N1—C3	90.66 (12)	C5—N1—C3—C4	−92.14 (18)
O2^i^—Ni1—N1—C3	177.11 (11)	Ni1—N1—C3—C4	30.37 (17)
O2—Ni1—N1—C3	−2.89 (11)	Ni1—O2—C4—C3	49.91 (15)
N1^i^—Ni1—N1—C3	98 (100)	N1—C3—C4—O2	−55.33 (19)
O1^i^—Ni1—O1—C2	29 (100)	C1—N1—C5—C6	63.6 (2)
O2^i^—Ni1—O1—C2	−91.17 (12)	C3—N1—C5—C6	−61.9 (2)
O2—Ni1—O1—C2	88.83 (12)	Ni1—N1—C5—C6	178.71 (13)
N1—Ni1—O1—C2	5.39 (12)	N1—C5—C6—O3	82.7 (2)
N1^i^—Ni1—O1—C2	−174.61 (12)		

Symmetry codes: (i) −*x*+1/2, −*y*+3/2, −*z*+2; (ii) −*x*+2, *y*, −*z*+3/2.

### Hydrogen-bond geometry (Å, °)

**Table:**

*D*—H···*A*	*D*—H	H···*A*	*D*···*A*	*D*—H···*A*
O1—H1C···O5^iii^	0.82	2.19	2.663 (2)	117
O2—H2C···O4^iv^	0.82	2.28	2.6227 (19)	106
O3—H3C···O5	0.82	2.00	2.818 (2)	174
C3—H3B···O3	0.97	2.26	3.055 (3)	139

Symmetry codes: (iii) *x*−1, *y*, *z*; (iv) −*x*+1, *y*, −*z*+3/2.

## References

[bb1] Fujita, M., Kwon, Y. J., Washizu, S. & Ogura, K. (1994). *J. Am. Chem. Soc.* **116**, 1151–1152.

[bb2] Haukka, M., Kirillov, A. M., Kopylovich, M. N. & Pombeiro, A. J. L. (2005). *Acta Cryst.* E**61**, m2746–m2748.

[bb3] İçbudak, H., Yilmaz, V. T., Howie, R. A., Andaç, Ö. & Ölmez, H. (1995). *Acta Cryst.* C**51**, 1759–1761.

[bb4] Kahn, O. (1993). In *Molecular Magnetism* New York: VCH.

[bb5] Kepert, C. J. & Rosseinsky, M. J. (1999). *Chem. Commun.* **1**, 31–32.

[bb6] Krabbes, I., Seichter, W. & Gloe, K. (2000). *Acta Cryst.* C**56**, e178. 10.1107/S0108270100004819 15263134

[bb7] Sheldrick, G. M. (1996). *SADABS* University of Göttingen, Germany.

[bb8] Sheldrick, G. M. (2008). *Acta Cryst.* A**64**, 112–122. 10.1107/S0108767307043930 18156677

[bb9] Siemens (1994). *SMART* and *SAINT* Siemens Analytical X-ray Instruments Inc., Madison, Wisconsin, USA.

[bb10] Topcu, Y., Andac, O., Yilmaz, V. T. & Harrison, W. T. A. (2001). *Acta Cryst.* E**57**, m82–m84. 10.1107/s0108270100019867 11250574

[bb11] Ucar, I., Yesilel, O. Z., Bulut, A., Icbudak, H., Olmez, H. & Kazak, C. (2004). *Acta Cryst.* E**60**, m322–m324. 10.1107/S0108270104013174 15295171

[bb12] Venkataraman, D., Gardner, G. B., Lee, S. & Moore, J. S. (1995). *J. Am. Chem. Soc.* **117**, 11600–11601.

[bb13] Yeşilel, O. Z., Bulut, A., Uçar, İ., İçbudak, H., Ölmez, H. & Büyükgüngör, O. (2004). *Acta Cryst.* E**60**, m228–m230. 10.1107/S0108270104020566 15467130

